# Geochemical baseline values and spatial distribution of major, trace, and rare earth elements in unpolluted soils of the Sicily region (Italy)

**DOI:** 10.1007/s10653-025-02475-z

**Published:** 2025-04-12

**Authors:** Federica Lo Medico, Daniela Varrica, Marino Vetuschi Zuccolini, Marianna Miola, Giovanna Scopelliti, Maria Grazia Alaimo

**Affiliations:** 1 Department Scienze della Terra e del Mare (DiSTeM), Università degli Studi di Palermo, Palermo, Italy; 2 Department Scienze della Terra, dell’Ambiente e della Vita (DISTAV), Università degli Studi di Genova, Genoa, Italy; 3 CNR-IMATI, Istituto “E.Magenes”, Genoa, Italy

**Keywords:** Soil, Regional geochemical baseline, Spatial distribution maps, Trace and ultra trace elements, Contamination level

## Abstract

This study presents a comprehensive investigation of the geochemical baseline values and the spatial distribution of major, trace elements, and rare earth elements in the unpolluted soils of Sicily region. The concentrations of elements were quantified by ICP-OES and ICP-MS. The distribution patterns of major and trace elements are closely linked to the geological features of the area. The major elements Fe, Ca, Al, Mg, and K exhibit the highest concentrations. The order of abundance for trace elements follows this trend: Ba, Sr, Zn, V, Cr, Cu, Rb, Ni, Pb, B, Li, Co, As, Mo, U, Sb, Se, Cd, and Bi. Regional geochemical baseline values were calculated using the UTL95 - 95 method with BCA bootstrap, demonstrating that the regional approach provides a more accurate assessment compared to European and Italian threshold values. A geostatistical approach was used to produce spatial geochemical maps, which allow the prediction of element distributions in unsampled areas. This integrated approach establishes a benchmark for more detailed studies on environmental risk assessment, providing a solid foundation for identifying and understanding natural and anthropogenic geochemical anomalies in the soils of the Sicily region.

## Introduction

Soil plays a crucial role in ecosystems as it is a dynamic habitat that supports a variety of biological processes. The interactions within soil contribute significantly to nutrient cycling, energy flow, and the overall health of ecosystems. The topsoil layer is of particular interest as degradation may occur due to atmospheric deposition, anthropogenic activities, and/or natural geochemical processes (US Aelion et al., 2008; EPA, 1992).

Major and trace elements occur naturally in soils, with their concentrations influenced by various factors. These include the physical and chemical weathering of parent rocks, pedogenesis, and the transport of materials through colluvial, fluvial, or even aeolian processes. Additionally, the mobility of each element, which varies according to specific physico-chemical parameters, plays a key role in determining their distribution. The concentrations of these in soils can be significantly modified by anthropogenic pressures of urban, industrial, mining and agricultural origin (Belon et al., 2012; Modaberi et al., 2023). Among the different categories of elements encountered, major, trace elements and rare earth elements (REEs) each play a distinct role in environmental health and toxicology. Major elements such as calcium, potassium, and magnesium, although generally essential, can also affect human health depending on exposure levels and biochemical interactions. Trace elements, which occur in minor quantities in natural systems, include essential elements like iron, zinc and copper, which are required in small amounts for biological function, and toxic elements such as arsenic, cadmium and lead, which pose risks even at low concentrations (Prashanth et al., 2015). Rare earth elements have recently attracted attention due to their increasing industrial use and the resulting potential risk to human health (Albanese et al., 2023; Cicchella et al., 2020).

Italian legislation (D. Lgs. 152/2006) regulates only a limited number of inorganic elements (As, Be, Cd, Co, Cr, Cu, Hg, Ni, Pb, Sb, Se, Sn, Tl, V, and Zn), therefore a proper geochemical investigation cannot ignore elements known to be potentially toxic, even if they are not considered hazardous according to national legislation. Therefore, in order to properly assess and monitor environmental quality, we analyze a wider range of inorganic elements. Recognizing the main elements of soil pools and the driving factors that can be ascribable to geogenic and/or anthropogenic sources is essential, as it is also necessary to evaluate background values ​​(Négrel et al., 2015; Reimann & de Caritat, 2005). The background value corresponds to the range of element concentrations in a specific area entirely reliant on the compositional and mineralogical features of the local rocks. However, even when defining background values ​​in unpolluted areas, it is impossible to exclude a global contribution related to atmospheric deposition that can introduce additional elements into topsoils (Santos-Francés et al., 2017). Considering this, it is necessary to introduce the term geochemical baseline as defined by the International Geological Correlation Program (Darnley et al., 1995). Determination of baseline values ​​in topsoil samples from unpolluted areas can provide a reference point that reflects current environmental conditions. These reference values ​​could be used as standards for assessing soil quality, implementing environmental protection measures, and supporting legislation to detect changes resulting from human activities (Cicchella et al., 2005; De Vivo et al., 2008; Gałuszka, 2007; Jarva et al., 2010; Salminen & Gregorauskiene, 2000; Xie & Ren, 1993).

To achieve a comprehensive understanding of the study area, it is essential not only to determine the geochemical baseline values but also to analyze the spatial distribution of elements. This approach enables the evaluation of their variability and the identification of areas with potentially hazardous concentrations through geochemical mapping. This integrated approach allows us to distinguish contaminated areas from non-contaminated ones, evaluate the current environmental state, and serve as a reference for future assessments (Baize & Sterckeman, 2001; López et al., 2008; Salminen et al., 2004; Tack et al., 2005; Tarvainen & Paukola, 1998; Wang et al., 2007).

In this study, the Sicily region, known for its significant lithological heterogeneity, was selected as a pilot site for the definition of reference values encompassing major, trace elements, and REEs in topsoils. The choice of the Sicily region, was determined by the lack of analytical data on the current environmental conditions of topsoils. Therefore, the methodological approach used represents the first attempt to define the regional geochemical baseline (Varrica et al., 2024).

The objectives of this research include the following:

- Establish baseline values: determine the natural concentrations of major, trace, and rare earth elements (REE) in unpolluted soils. Provide regional geochemical baseline values ​​through a statistical approach.
- Spatial distribution mapping: analyze and map the spatial variability of these elements in the study area and predict the distribution of concentrations of each element, even in unsampled areas.

## Materials and methods

### Sampling and analytical methods

A total of 83 topsoil samples (depth of 0–20 cm) were collected far from anthropogenic sources (i.e. extra-urban roads, urban areas, industrial areas, cultivated land) in the Sicilian territory. The sampling plan design envisaged the total coverage of the different lithological units of Sicily by collecting 65 soils in the sedimentary substrate, 8 in the volcanic substrate, and 10 in the metamorphic substrate.

**Fig. 1:**
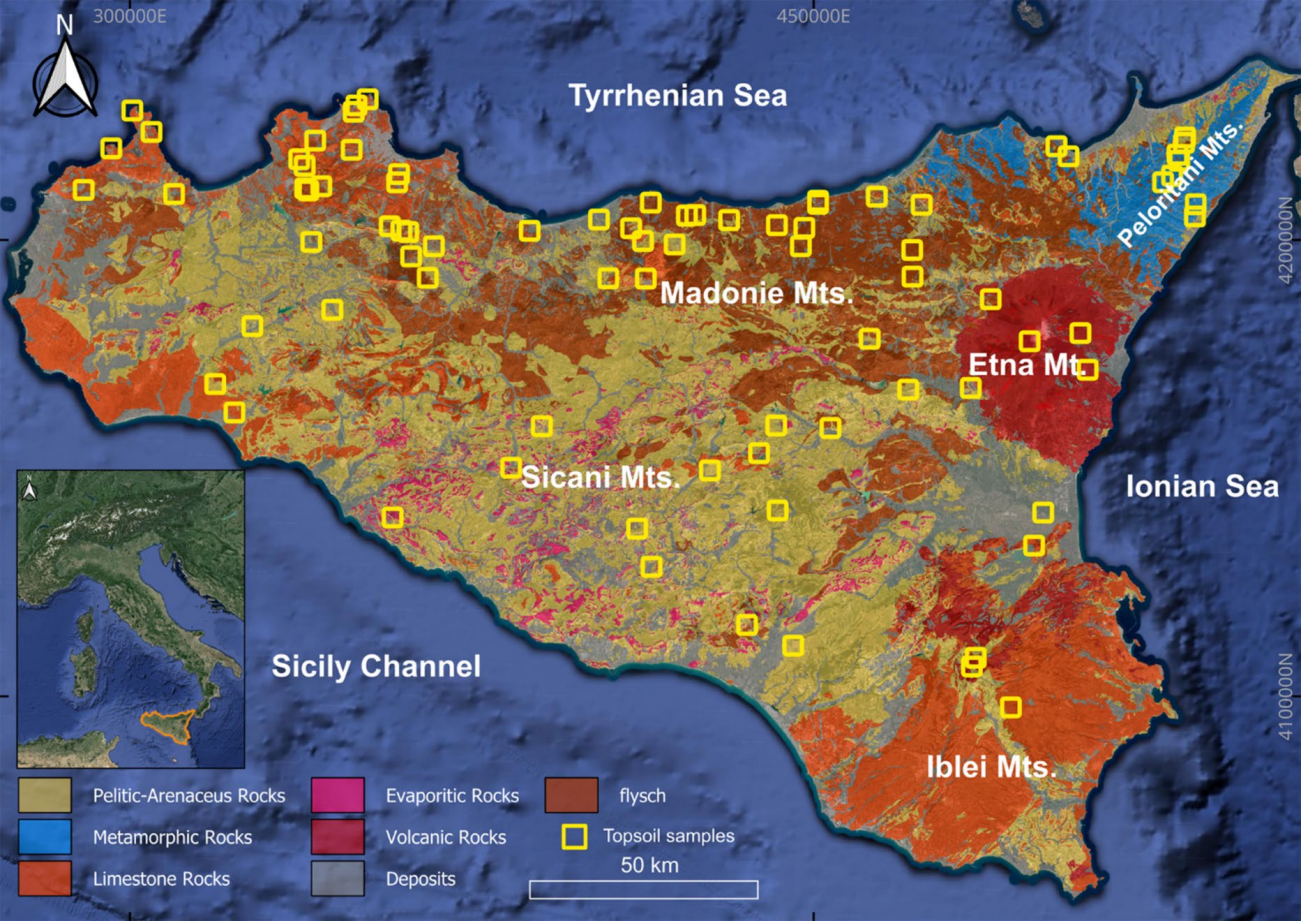
Distribution topsoil samples and main geological domains of Sicily region (modified from Catalano et al., 2013)

The distribution of the samples is shown in Fig. 1. The study area is characterized by a Mediterranean climate marked by hot, dry summers and mild, wet winters (Kottek et al., 2006). Geologically, the study area shows an extremely complex and diverse structure linked to a series of tectonic events which are reflected in the great lithological variety of the rocks that outcrop (Catalano et al., 2013). In the northern part, carbonate rocks predominate. On the eastern side, outcrops of clayey sandstones and metamorphic rocks are found. The southeastern part consists mainly of carbonate and clastic rocks, with some intercalations of basic volcanic rocks. The central part of the island is characterized by syn- and post-orogenic deposits, the Gessoso-Solfifera Formation, sandy calcarenites, and marly limestones. The area surrounding Mount Etna is characterized by rocks ranging in composition from basaltic to trachytic. However, most of Mount Etna’s lava flows are of hawaiitic composition, as described by Liguori and Brucculeri (2004).

The samples were collected using a plastic scoop, stored in PVC packages, and transported to the laboratory. They were dried at 105 °C in a stove (AG System, mod. G-therm) overnight; stones and plant materials were removed, and each sample was sieved using 500 μm nylon mesh, then milled in agate mortar. Soil analysis was carried out at an accredited laboratory “Activation Laboratories Ltd. (Ontario, Canada)”. The topsoil samples were digested with a mixture of 3 ml hydrochloric acid and 1 ml of nitric acid. The samples were analyzed for major (Ca, Fe, K, Mg, Mn, Na, P, Sr and Ti), trace (B, Ba, Bi, Co, Li, Ni, Rb, Se, U, and REE elements (La, Ce, Pr, Nd, Sm, Eu, Gd, Tb, Dy, Ho, Er, Tm, Yb, Lu) by ICP-OES and ICP-MS. The recovery rates of certified elements in the reference standard materials (OREAS- 45 d, OREAS- 922, OREAS- 907, OREAS- 263, OREAS − 130, OREAS- 521, OREAS- 620, OREAS- 610) were 96–112% for major and trace elements; for REEs, the recovery was 94–120%. Analytical precision, estimated from duplicate analyses every tenth sample was in the range of 1–7% for all analyzed elements, excluding samarium and thulium in which the range was 11–14%. The concentration data of Al, As, Cd, Cr, Cu, Mo, Pb, Sb, V, and Zn used in this study were taken from the literature (Varrica et al., 2024). The mineralogical characterization of the soils was determined semi-quantitatively by X-ray powder diffraction (XRD) using a Philips PW14 1373 with Cu-Kα radiation of the Department of Scienze della Terra e del Mare (DiSTeM), University of Palermo. The measurement of pH values was performed potentiometrically on the soil suspensions obtained by adding 25 ml of potassium chloride 1 M to 10 g of soil (dry weight). For the semi-quantitative determination of total organic carbon (TOC), the sample dried at 105 °C overnight is weighed, and the loss on fire (LOI) is performed, heating it to 450 °C, in a muffle furnace for 6 h. The percentage of LOI, calculated re-weighing the samples, is the expression of the organic matter content, therefore a conversion factor of 1.724 has been used to convert it into total organic carbon (Nelson & Sommers, 1996).

### Data analysis

Data were analyzed statistically, and all tests were considered significant at p < 0.05 using the software XLSTAT, and ProUCL 5.1 software (Singh & Maichle, 2015). The normality of data distributions was assessed using the Shapiro–Wilk test (W). The results indicate that the distribution of the analyzed elements is non normal. Among the dispersion parameters, the robust coefficient of variation (%rCV) was used (Ambrosino et al., 2023). This was calculated as follows: %rCV = MAD/Median × 100, where MAD is the Median Absolute Deviation. An R-mode cluster analysis was conducted to examine the relationships between elements and identify potential distinct groups within the data. The clustering procedure was performed by the unweighted pair–group average method (UPGM) with the Spearman coefficient as a measure of similarity of elements (*p* < 0.05).

### Determination of regional geochemical baseline (RGB)

Regional Geochemical Baseline (RGB) values for major, trace elements and REEs were determined using a statistical approach. As highlighted by Varrica et al., 2024, for the Sicily region the UTL95-–95 BCA Bootstrap method (SNPA, 2018) is the most suitable. This approach was chosen for its ability to provide robust and reliable estimates. The baseline value is calculated using the ProUCL 5.1 software, a tool designed for the statistical analysis of environmental data, capable of adapting to various types of distributions. In the case of non-normal distributions, the software indicates the non-parametric UTL95 - 95 value as the reference value. UTL95 - 95 corresponds to the Upper Tolerance Limit at the 95% level (UTL) with a 95% coverage (UTL95 - 95). The BCA bootstrap method (Efron & Tibshirani, 1993; Manly, 1997) is used in combination with UTL95 - 95 to further improve the estimation of the upper tolerance limit (UTL) and correct for any distortions in the data. Bootstrap is a statistical technique that allows the creation of repeated and simulated samples (called “bootstrap samples”) from the original data, enabling a more accurate estimate of the parameters of interest. Once the bootstrap samples are created, a bias correction is applied. Bias represents any systematic distortion that could affect the results, and the bias correction serves to remove these distortions. Finally, data acceleration is an additional step that is carried out to improve the estimation of confidence intervals. Acceleration accounts for the variability in the data and helps refine the estimates. Overall, the combined use of the BCA bootstrap method and the UTL95 - 95 value, supported by ProUCL 5.1 software, provides a solid foundation for the analysis and interpretation of geological and environmental data.

### Contamination indicators

The *Geo-accumulation Index* (I_geo_) is used to assess the accumulation of chemical elements in sediments or soils over time. The I_geo_ is calculated from Eq. (1) (Suresh et al., 2012):

1 $${\text{I}}_{{{\text{geo}}}} = {\text{ Log}}_{{2}} \left[ {{\text{C}}_{{\text{i}}} /\left( {{1}.{\text{5B}}_{{\text{i}}} } \right)} \right]$$

where C_i_ is the measured concentration of the i-th chemical element examined in the topsoil sample, and B_i_ is the baseline level of the i-th chemical element. The baseline values used in the contamination indicators are those calculated in this study. Factor 1.5 was used to correct possible variations in the background values of a particular elements in the environment (Santos-Francés et al., 2017). According to Müller classification (1969), the I_geo_ consists of 7 classes: uncontaminated (I_geo_ ≤ 0), uncontaminated to moderately contaminated (0 < I_geo_ ≤ 1), moderately contaminated (1 < I_geo_ ≤ 2), moderately to heavily contaminated (2 < I_geo_ ≤ 3), heavily contaminated (3 < I_geo_ ≤ 4), heavily to extremely contaminated (4 < I_geo_ ≤ 5) and extremely contaminated (I_geo_ > 5).

The *Enrichment Factor (EF)* is defined by Yongming et al. (2006) as Eq. (2):

2 $$EF = \, \left( {Ref/X} \right)_{sample} /\left( {Ref/X} \right)_{baseline}$$

where X represents the element of interest and Ref the reference element respectively in the sample (numerator) and in the calculated regional baseline (denominator). In this study, we used aluminum as a reference element based on these considerations: (1) it is high natural abundance, (2) it is considered as a conservative element undergoing minimal disturbance by biological, diagenetic, and redox processes (Rollinson, 2014), and (3) it is easily determined by conventional techniques. The categories based on Enrichment Factor (EF) were (Yongming et al., 2006): EF < 2 Deficiency to minimal enrichment; 2 < EF < 5 Moderate enrichment; 5 < EF < 20 Significant enrichment; 20 < EF < 40 Very high enrichment; EF > 40 Extremely high enrichment.

The *Contamination Factor* (CF) is the ratio obtained by dividing the element concentration in the investigated site by the background values (Rutkowski et al., 2020).

The CF was calculated using Eq. (3):

3 $${\text{CF }} = {\text{ C}}_{{\text{m}}} /{\text{C}}_{{\text{b}}}$$

where C_m_ is the concentration of an element measured in soil sample and C_b_ is the baseline level of the element calculated in this study The contamination factor of each metal was classified as follows Rutkowski et al., 2020: CF 0–1 No contamination; CF > 1–2 Suspected contamination CF > 2–3.5 Slight contamination CF > 3.5–8 Moderate contamination CF > 8–27 Severe contamination CF > 27 Extreme contamination.

The *Pollution Load Index* (PLI) originally proposed by Tomlinson et al. (1980) was also used to evaluate for the entire sampling site by the calculation of the product of the nCF (Eq. 3) (Usero et al., 2000) follows Eq. (4)

4 $${\text{PLI }} = \, \left( {{\text{CF}}_{{1}} \times {\text{ CF}}_{{2}} \times {\text{ CF}}_{{3}} \times {\text{ CF}}_{{4}} \times \, . \, . \, . \, \times {\text{ CF}}_{{\text{n}}} } \right)^{{{1}/{\text{n}}}}$$

where CF is a contamination factor and n is number of studied elements. The PLI was classified as follows: PLI < 0.9 unpolluted area, a PLI approaching 1 indicates a pollution load close to the background level, 1.1 < PLI < 1.5 low pollution, 1.5 ≤ PLI < 2.0 moderate pollution, PLI ≥ 2.5 very high pollution.

### Geochemical mapping

The regional dataset consists of 83 samples collected in a heterogeneous geological area. The samples are distributed in clusters with redundant sampling sites. Stochastic simulations (Deutsch & Journel, 1998; Gómez-Hernández & Srivastava, 2021) were used to generate the spatial distribution maps of elemental contents in soils instead of deterministic interpolation strategies such as B-spline, low-pass filtered, Inverse Distance Weighting (IDW) or Kriging algorithms (Cressie, 1990). The latter typically produce high continuity results that are prone to artefacts, whereas the former are based on the spatial covariance laws (Goovaerts, 1997), which allow to take into account the short-scale variability and to assess the local uncertainty associated with the estimates.

In this context, stochastic simulations have been used to calculate the spatial distributions on a convex-concave boundary at a resolution of 5 km in order to cover the entire region of Sicily.

The limited number of samples used precluded using the same segmentation as that used to calculate the baseline. Therefore, estimates were made for the whole area (N. 1067 cells). Each cell, further divided into four subcells, was associated for 50 iterations. The simulation process involved anamorphosis to a normal standard distribution and then back-transformation of the (normal) estimates to the original space, which served to reduce bias due to the heterogeneous sampling scheme and the asymmetric probability distribution function of the input data. The distribution maps were elaborated for the selected elements using the program QGIS 3.34.6 Prizren (2023).

## Results and discussion

The parameters that characterize soils, pH and organic matter content, are closely associated with soil formation (Jenny, 1941; Slessarev et al., 2016). The pH values ​​determined in soil samples show a wide variability related to the underlying bedrock (Fabian et al., 2014; Gruba & Socha, 2016; Reuter et al., 2008), ​​ranging from 5.9 to 7.2. The results of the TOC test (Storer, 1984) highlight that 72% of the samples have a high amount of organic matter (> 5%), followed by 22% with an organic matter range between 2 and 4%, classified as medium. The main minerals determined in the topsoil samples by the diffraction analysis highlight significant peaks of calcite, plagioclase, and illite, followed by quartz, clinopyroxene, kaolinite, muscovite, and chlorite. In lower quantities, smectite has been detected. This mineralogical characterization reflects the heterogeneity of the types of soils and the different geological environments of formation.

### Major and trace elements

Table 1 reports the main statistical parameters of the elements analyzed on the surface soil samples. The non-overlapping mean and median values ​​suggest an asymmetric distribution for all the determined elements, as confirmed by the Shapiro–Wilk normality test (*p* < 0.05). The high value of the skewness index (> 1.5) for all analyzed elements, except for Cr and Zn, indicates an extremely right-skewed distribution. The values of the kurtosis coefficient indicate that the distributions have heavier tails compared to a normal distribution, suggesting a leptokurtic distribution for all elements, except for B, Ca, Co, Cr, Fe, Mn, Se, and Zn that show platykurtic distributions. Concentration patterns of the major and trace elements reflect the geological features of the studied area. The higher values ​​of Fe, Ca, Al, Mg and K (ranging from 26,300 to 3500 µg g⁻^1^) are related to the underlying geology, which varies between metamorphic, volcanic, evaporite, limestone and clay rocks. In the group of trace elements, Ba, Sr, and Zn exhibit the highest concentrations, with values of 111, 77.5, and 68.8 µg g⁻^1^, respectively, followed by V, Cr, Cu, Rb, Ni, Pb, B, Li, Co, As, Mo, U, Sb, Se, Cd, and Bi. The order of abundance of trace elements is mainly influenced by the geological characteristics of the studied area. Table 1 also reports the %rCV which serves as a robust measure of relative dispersion. Lower robust coefficient of variation values ​​indicates greater stability. Conversely, higher %rCV values suggest that the MAD is relatively large compared to the median, indicating greater dispersion in element concentrations. The robust coefficient of variation calculated for major elements showed wide variability for all elements (> 30%) as defined by Ambrosino et al. (2023), except for Na (11%) and Mg (28%). The same wide dispersions were found for trace elements except for Sr (19%) and B (30%) highlighting the lithological heterogeneity of the study area.

**Table 1:** Statistics of major and trace element content in topsoil samples

		Median	MAD	Range	%rCV	Skewness	Kurtosis
Major	Al*	19,400	6300	68–91,005	31	2.08	6.56
	Ca	23,300	19,420	640–319,000	32	1.72	1.89
	Fe	26,300	9300	50–79,500	35	0.48	0.84
	K	3500	1900	100–29,789	40	3.06	12.44
	Mg	5700	3100	500–132,000	28	4.53	20.3
	Mn	578	243	142,628	37	1.13	1.72
	Na	320	150	50–24,632	11	5.47	37.1
	P	550	270	20–3330	38	1.87	4.37
	Ti	540	450	20–8940	42	2.79	9.72
Trace elements	As*	4.50	1.9	0.17–113	42	6.24	45.2
	B	16.0	10.5	5.0–57.0	95	1.21	1.52
	Ba	111.0	40.7	4.05–800	30	3.20	12.7
	Bi	0.21	0.1	0.03–2	53	3.44	17.0
	Cd*	0.23	0.08	0.04–4.30	35	4.02	21.3
	Co	9.70	5.3	0.46–34	55	0.60	0.25
	Cr*	28.0	13	3.64–94	46	0.97	1.06
	Cu*	25.7	13.7	3.91–130	53	1.81	3.87
	Li	14.0	6.4	1.37–59.4	52	1.71	3.80
	Mo*	1.16	0.39	0.40–12.48	34	4.54	26.1
	Ni	20.9	10.5	4–111	50	2.14	9.20
	Pb*	16.5	7.19	1.55–111	44	2.78	12.4
	Rb	22.9	10.9	1.50–180	48	3.01	13.0
	Sb*	0.40	0.18	0.07–7.75	45	7.56	63.9
	Se	0.40	0.25	0.05–1.6	83	1.05	1.33
	Sr	77.5	50.3	11.13–4050	19	4.82	27.5
	U	0.80	0.4	0.04–40	50	4.52	20.3
	V*	40.0	20	1–238	50	2.23	8.49
	Zn*	68.8	28.9	0.50–251	42	1.05	2.26

Concentration data expressed in µg g^−1^

MAD: Median Absolute Deviation; %rCV: robust coefficient of variation

*Data published in Varrica et al. (2024)

**Fig. 2:**
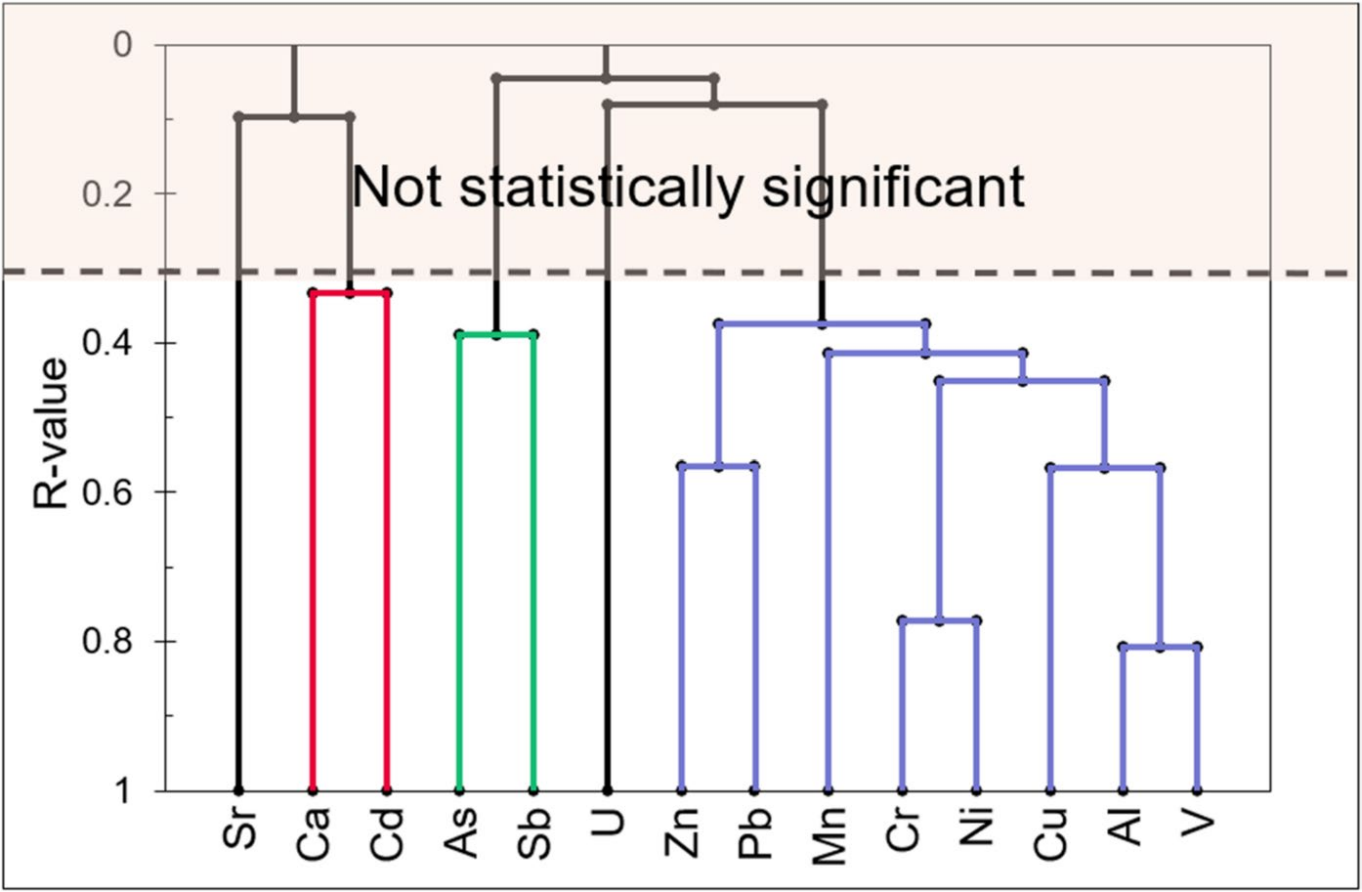
Dendrogram produced by cluster analysis (UPGM) for 83 samples. R values on the ordinant scale indicate the similarity of geochemical variables

A useful method employed to simplify the complex data set, with the aim of identifying relationships between variables, is R-mode cluster analysis. The clustering procedure was performed with Spearman’s coefficient as a similarity measure. A cluster analysis was performed using concentration data of 14 elements from the 28 available. We selected elements with no analytical determinations below the limit of detection and others with statistically significant correlation with the elements studied. Results are displayed in the bidimensional hierarchical diagram of Fig. 2. Three main groups of related elements may be identified at R > 0.3 (*p* < 0.05). Elements with loadings exceeding 0.3 are considered the most representative in defining the composition of each group. The clusters that were found reflect the geological characteristics of the investigated area. The group with high bonds of elements such as Al, Cr, Cu, Mn, Ni, Pb, V and Zn represents the underlying bedrock, identifiable with silicate rocks in a metamorphic-volcanic environment. Previous studies in the Etna area and the Peloritani mountains confirm the association of these trace elements (Aiuppa et al., 2000; De Vivo et al., 1993). The cluster As–Sb is attributed to polymetallic mineralizations containing As and Sb sulfides present in metamorphic rocks of Peloritani Mountains (De Vivo et al., 1993; Dongarrà et al., 2009; Ferla & Omenetto, 2000). The last cluster comprising Ca–Cd is attributed to a carbonate sedimentary environment (Giacalone et al., 2005; Kubier et al., 2019; Rambeau et al., 2010).

### Rare earth elements

Rare Earth Elements (REEs) are present in minerals in varying concentrations (Chakhmouradian & Wall, 2012; Jordens et al., 2013; Ramos, et al., 2016), and their availability in the environment under natural conditions depends on the source material, geochemical and biological processes (Cidu et al., 2013; Zhang et al., 2001). Table 2 presents the descriptive statistics of REEs in topsoil samples. The median of the total REEs content (ΣREEs from La to Lu) in soils analyzed is 85.8 µg g^−1^, ranging between 12.3 and 225.7 µg g^−1^. From Table 2, it is observed that the median of the individual REEs concentrations in the surface soils decreases in the following order: Ce > Nd > La > Pr > Gd > Sm > Dy > Er > Yb > Eu > Tb > Ho > Tm > Lu. Ce, Nd, and La are the most abundant REEs in the surface soils, with 31.3 µg g^−1^, 15.8 µg g^−1^, and 14.2 µg g^−1^ median contents, respectively. The individual REEs content for the remaining elements decreases significantly to median values ​​ranging from 4.3 µg g^−1^ for Pr to 0.1 µg g^−1^ for Lu. The robust coefficient of variation calculated showed wide variability for almost all REEs (> 30%) showing the lithological heterogeneity of the study area. The values of the LREE/HREE ratio, calculated is 9.51 indicating a consistent pattern of preferential enrichment of LREEs and depletion of HREEs (Chen & Yang, 2010; Ion & Cosac, 2023). Given that the topsoil samples were taken from areas remote from pollution sources, the observed degree of enrichment or depletion is entirely influenced by the compositional and mineralogical characteristics of the parent material. Ambrosino et al. (2022), in a study carried out on soils of the Campania region, found median values ​​of La and Ce higher than the threshold values ​​of European agricultural soils, confirming the exclusive dependence of the concentrations from bedrock identified in volcanic areas of Vesuvius and Campi Flegrei.

**Table 2:** Statistics of rare earth elements content in topsoil samples

Rare earth elements				
	Median	MAD	Range	%rCV
La	14.2	5.55	0.17–59.9	38
Ce	31.3	13.4	1.00–111	40
Pr	4.30	1.55	0.60–10.5	39
Nd	15.8	6.10	1.67–58.0	36
Sm	3.00	1.05	0.03–9.80	31
Eu	0.70	0.20	0.03–2.30	29
Gd	3.30	0.90	0.60–6.90	29
Tb	0.40	0.10	0.05–1.50	25
Dy	2.30	0.45	0.50–5.40	21
Ho	0.40	0.10	0.05–1.00	25
Er	1.00	0.30	0.20–2.60	30
Tm	0.10	0.05	0.03–0.40	50
Yb	0.80	0.20	0.05–5.70	29
Lu	0.10	0.05	0.02–0.80	50
	∑REE	∑LREE	∑HREE	∑LREE/∑HREE
	85.8	77.9	8.75	9.51

Concentrationdata expressed in µg g^−1^

MAD: Median Absolute Deviation; %rCV: robust coefficient of variation

### Regional geochemical baseline (RGB) and contamination level

The comparison of the median values ​​of the soils of the Sicily region with the elements regulated by the Italian law for soils (D. Lgs. 152/2006), as reported in Table 3, offers several matters for reflection. The values are all below the threshold values defined by law, confirming that the soil samples were collected in environments far from sources of pollution. Italian law does not consider all the inorganic elements, only those considered toxic. Instead, it would be appropriate to know reference thresholds for all the elements that perform functions of nutrients, micronutrients, and potentially toxic. Furthermore, investigating the total distribution of concentrations in the samples, it is observed that the percent of samples that exceed the legislative thresholds for As and Co are approximately 5%, for V and Zn greater exceedances are observed between 6 and 8%. The percentages of samples that exceed the legislative contamination threshold values ​​reflect the geological heterogeneity of the study area. Previous studies have shown that soils exceeding contamination threshold concentrations defined by D. Lgs.152/2006 may not pose a risk to human health when the enrichment originates naturally (Ambrosino et al., 2024; Zuzolo et al., 2020).

**Table 3:** Comparison between the median values of Sicilian soils and the elements regulated by Italian legislation (D. Lgs 152/2006)

	As*	Cd*	Co	Cr*	Cu*	Ni	Pb*	Sb*	Se	V*	Zn*
Median	4.50	0.23	9.70	28.0	25.7	20.9	16.5	0.40	0.40	40.0	68.8
CSC (D.Lgs. 152/2006)	20	2	20	150	120	120	100	10	3	90	150
% of samples above of CSC	4.8	2.4	4.8	0	2.4	0	1.2	0	0	8.4	6.0

Concentrationdata expressed in µg g^−1^

CSC: Contamination Threshold Concentration

*Data published in Varrica et al. (2024)

Research has documented cases of naturally high concentrations surpassing contamination thresholds in volcanic and metamorphic soils (Varrica et al., 2024). Therefore, it is necessary to calculate threshold values ​​that are representative of the regional geology of the study area, to obtain a more accurate indication in the determination of environmental contamination indices. Among the different methods used to calculate geochemical baseline values, the 95 th percentile and the UTL95 - 95 method are worth mentioning. The 95 th percentile is easier to determine but less robust to data bias. At the same time, the UTL95 - 95 method with BCA bootstrap is more robust as it takes into account variability and bias in the data. Furthermore, the UTL95 - 95 method is recommended by the U.S. Environmental Protection Agency (US EPA) to establish threshold values since, among the applied methods, it is the most suitable for evaluating upper limits in quality control and environmental monitoring contexts. This study applied the UTL95 - 95 method calculated using the ProUCL 5.1 software (Table 4) to determine Regional Geochemical Baseline (RGB) values. From Table 4, it is evident that the calculated values exceed the median values while remaining below the national legislative thresholds, with the exceptions of cobalt and vanadium. The calculated RGB value for cobalt (22 µg g⁻^1^) slightly exceeds the legislative threshold (20 µg g⁻^1^), while the value for vanadium (88.2 µg g⁻^1^) is close to the legislative threshold (90 µg g⁻^1^). In the same Table 4, the EU guideline value ranges (Reimann et al., 2014) ​​and the threshold values ​​defined by a European study-GEMAS (Reimann et al., 2018) are reported for comparison purposes. All RGB values ​​are lower than the Italian and GEMAS regulatory limits, while compared to the guideline value ranges provided by the European Community, our data are close to the lowest values ​​of the range. This highlights that the regional approach offers more specific and significant indications than the European and Italian reference values. However, this approach may result in the underestimation or overestimation of certain elements when calculating contamination status (Varrica et al., 2024).

**Table 4:** Comparison of Regional Geochemical Baseline values (RGB) for major, trace elements and rare earth elements with to the Italian legislation (D. Lgs 152/2006), EU range guideline values ​​(Reimann et al., 2014) and the threshold values defined by a European study—GEMAS (Reimann et al., 2018)

	RGB	IT D.Lgs	GEMAS	Range EU		RGB	IT D.Lgs	GEMAS	Range EU		RGB	GEMAS
Al*	41,414				Mn	1303		1415	1500	La	39.3	32
As*	11.8	20	21	10–200	Mo*	3.96		1.7	2.5–60	Ce	77.0	
B	33.2		10	5–1000	Na	4748				Pr	7.36	
Ba	245.9		180	100–600	Ni	44.89	120	66	30–300	Nd	36.7	
Bi	0.67		0.49	0.5–1	P	1335				Sm	7.06	
Ca	186,000				Pb*	41.3	100	44	40–750	Eu	1.55	
Cd*	1.16	2	0.61	0.5–20	Rb	53.6				Gd	4.90	
Co	22.0	20	22	20–100	Sb*	1.14	10	1.00	10–50	Tb	0.70	
Cr*	58.1	150	70	30–1000	Se	1.00	3	0.88	1–20	Dy	3.60	
Cu*	62.9	120	50	40–1000	Sr	762				Ho	0.61	
Fe	52,007				Ti	2830				Er	1.50	
K	11,617				U	2.80		3.4	20	Tm	0.10	
Li	30.6		33		V*	88.2	90	63	100–220	Yb	2.31	
Mg	18,119				Zn*	124	150	104	60–2500	Lu	0.30	

*Data published in Varrica et al. (2024)

Furthermore, in this study, the baseline values ​​were also calculated as for elements considered not potentially toxic, as for technology-critical elements (TCEs) that have significance in the environmental studies.

**Fig. 3:**
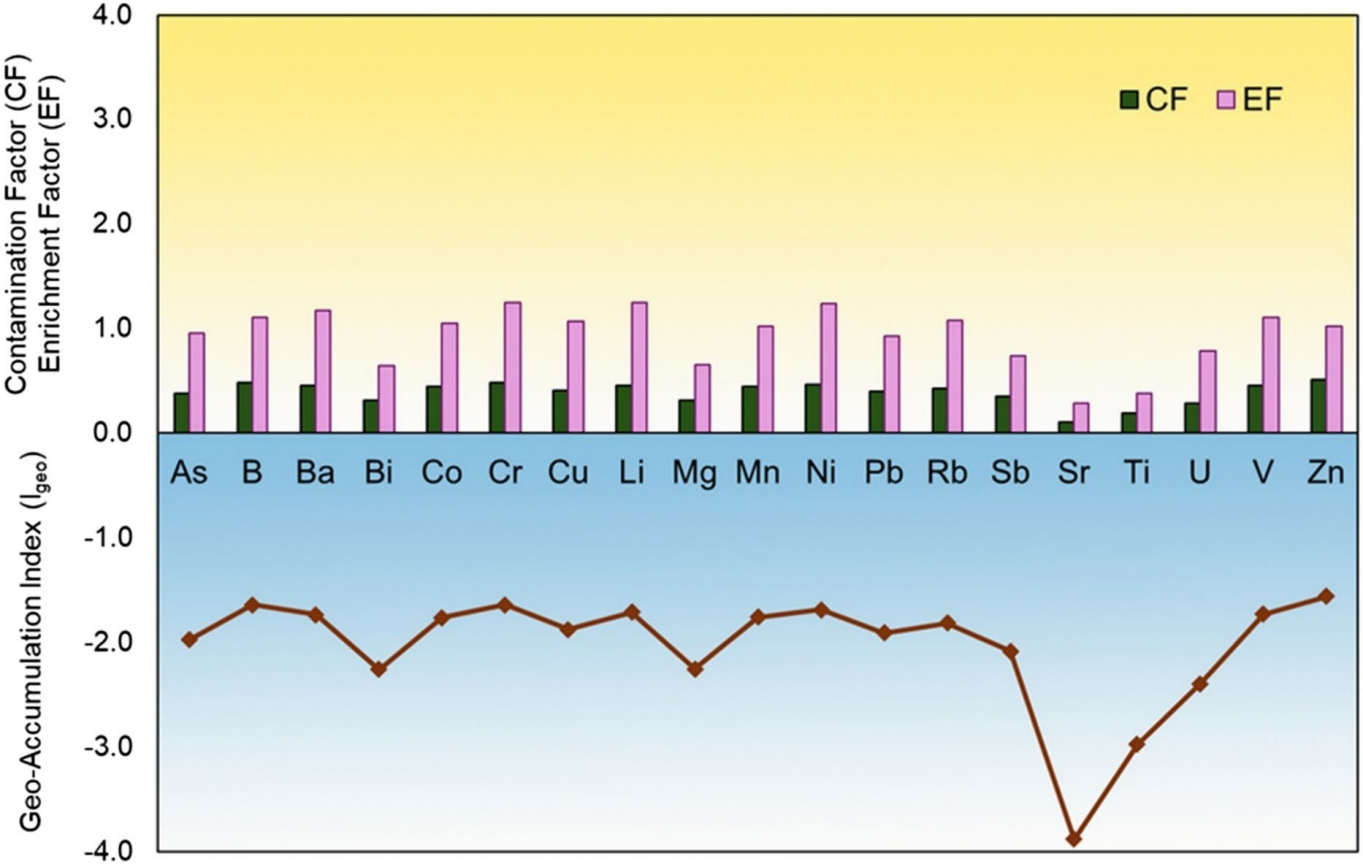
Contamination indices trends for: **a** Enrichment factor (EF) and Contamination factor (CF); **b** Geoaccumalation index (I_geo_)

The contamination status of the topsoil layer was assessed by comparing of the several contamination indicators as: Enrichment factor (EF), Contamination Factor (CF), Geoaccumulation index (I_geo_) and Pollution Load Index (PLI). All indices were calculated for all samples using the regional geochemical base values ​​(RGB) as a reference value. Previous studies (Sappa et al., 2020; Sierra et al., 2015; Yongming et al., 2006) have highlighted the need to use the local baseline values to define the soil contamination status. Figure 3 illustrates the values ​​of the indices calculated for the elements of toxicological and technological interest. An analysis of the individual indices reveals that the EF for all the elements considered is consistently below 2, which, according to the classification by Yongming et al. (2006), indicates a status categorized as “Deficiency with minimum enrichment”; the CF values range between 0 and 1 for all elements, corresponding to a “Low contamination factor” in according to the classification of Rutkowski et al. (2020). The Pollution Load Index (PLI) calculation yields a value of 0.37, confirming the non-contaminated status of the sampled sites (Usero et al., 2000). Finally, the I_geo_ for all elements shows values below 0, further validating that the topsoil samples are uncontaminated (Suresh et al., 2012).

### Geochemical spatial distribution

**Fig. 4:**
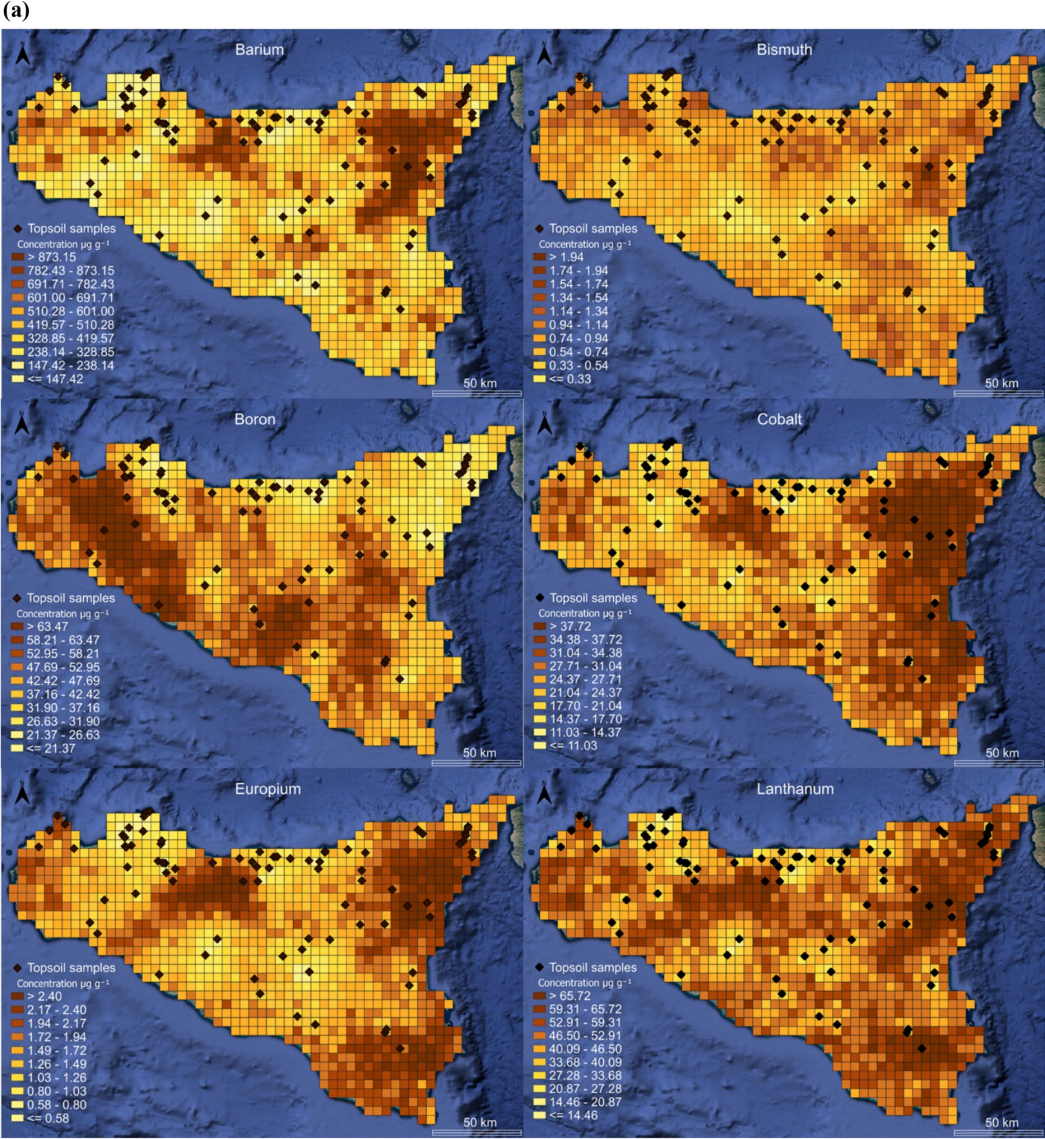

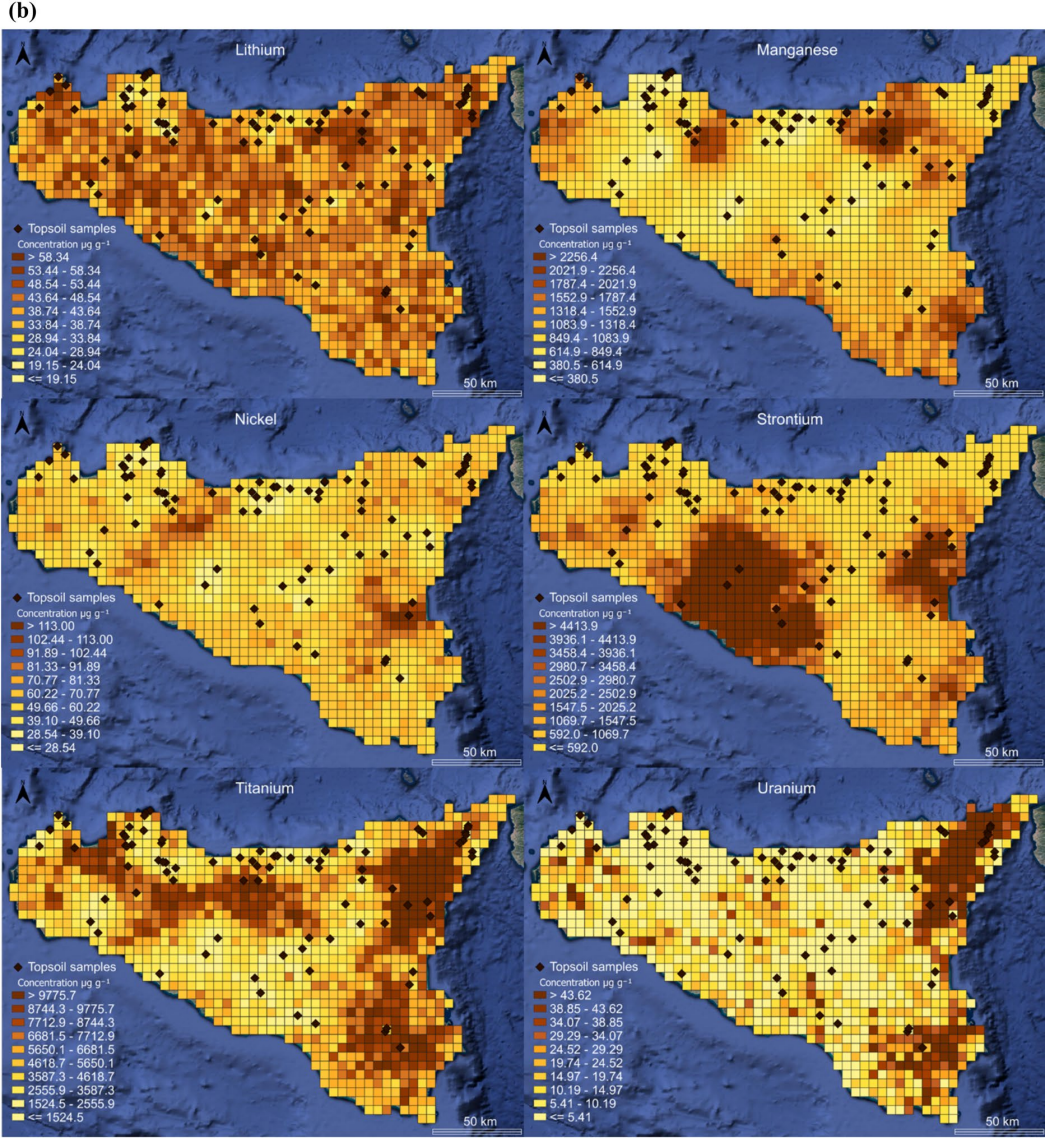
Distribution map of B, Ba, Bi, Co, Eu, La, (**a**) and Li, Mn, Ni, Sr, Ti, U (**b**). Data expressed in µg g^−1^

In order to obtain the spatial distribution of the concentrations of the analyzed elements, geochemical maps were generated. A geostatistical method was used to predict the spatial distribution of the concentrations of each element in unsampled areas based on the values of the collected samples. The numerical distribution is based on a probabilistic approach using stochastic simulations. The development of a regionally extended numerical tool is closely related to the quantification of baseline values. The applied numerical model reports the spatial statistical distributions of the following elements: B, Ba, Bi, Co, Eu, La, Li, Mn, Ni, Sr, Ti, and U. These distributions are the result of the stochastic process performed with over 50 simulations for each selected element. Considering the subdivision into sub-cells, each cell of the spatial distribution model contains 200 values. Based on this dataset, it is possible to construct a cumulative probability distribution function, from which the statistical moment corresponding to the 95 th percentile (P95) is extracted. P95, often chosen as a relevant threshold to identify extreme values as potential evidence of geochemical anomalies, is appropriately represented in Fig. 4a and b. The spatial geochemical distribution maps of individual elements in the topsoil provide valuable information about their concentrations, distributions, and origins. These maps are essential for identifying strongly site-specific geochemical baseline values. By mapping the distribution of elements, it is possible to identify regions with elevated concentrations that may indicate mineralization zones or naturally enriched areas.

Visualization of monoelemental maps for elements like B, Co, Sr, Ti and U, displayed a clear demarcation between the soils developed on different lithologies (sedimentary, volcanic, and metamorphic). The highest Co, Ti, and U concentrations were found in areas of the Sicily region characterized by volcanic-basaltic rocks (Mt. Etna) and metamorphic rocks (Mt. Peloritani) (Alexei & Beus, 1971; Carr & Turekian, 1961; IAEA, 1989). Regarding elements such as B and Sr, anomalies are found in central-western Sicily, strictly related to the outcrops of the Gessoso-Solfifera Series (Helvaci et al., 1993; Rossel et al., 1998). Regarding Ti and Eu, their distribution highlights the strong influence of the silicate rocks present in eastern Sicily and the influence related to the flyschoid rocks (Aide & Aide, 2012; Force, 1976). This overview underlines the strong influence of the parent material on the soil chemistry since the mineralogical composition of the bedrock contributes directly to the elemental composition of the overlying soils and suggests that the parent materials play a decisive role in the distribution of soil elements (Dantu, 2014). These observations highlight the importance of a detailed mapping of the elemental distribution to identify naturally enriched areas, in which the concentrations of specific elements are significantly higher than both the Contamination Threshold Concentrations (CSC) and the calculated regional geochemical baseline values. A systematic analysis of geochemical variability allows not only to distinguish natural anomalies from potential anthropogenic contamination, but also to understand the geological and geochemical processes that have determined such enrichments.

## Conclusions

The use of the geochemical baseline values and spatial distribution of elements in soils will deliver useful input to distinguish natural from contaminated soil composition. This study provides the first results of the regional distribution of major (Ca, Fe, K, Mg, Mn, Na, P, Sr and Ti), trace (B, Ba, Bi, Co, Li, Ni, Rb, Se, U), and rare earth elements (La, Ce, Pr, Nd, Sm, Eu, Gd, Tb, Dy, Ho, Er, Tm, Yb, Lu) in the unpolluted soils of Sicily region. Elemental associations obtained through R-mode cluster analysis have been very helpful in interpreting and distinguishing data by lithogenic sources. The region is dominated by the association of elements such as Al, Cr, Cu, Mn, Ni, Pb, V, and Zn, representing the underlying bedrock, identifiable with silicate rocks in a metamorphic-volcanic environment. The cluster As–Sb is attributed to polymetallic mineralizations containing As and Sb sulfides in methamorphic rocks, and the cluster comprising Ca–Cd is attributed to a sedimentary environment. The values of the regional geochemical baseline were determined using a statistical approach by selecting the UTL95 - 95 value calculated using the ProUCL 5.1 software. The matching between the elemental distribution and the regional geological features gives to geochemical maps a predictive character. The determination of geochemical baseline values and the assessment of spatial distributions provide essential support to local authorities in establishing reference points to adequately assess any present and future contamination in the soils of the Sicily region. This confirms that the statistical approach, even when applied to a limited number of samples, provides reliable results even in areas not covered by sampling.

Moreover, human health risk assessment, in conjunction with geochemical studies, helps to protect communities from adverse effects associated with soil contamination, ultimately contributing to safer environmental management practices.

## Data Availability

No datasets were generated or analysed during the current study.

## References

[CR1] Aelion, C. M., Davis, H. T., McDermott, S., & Lawson, A. B. (2008). Metal concentrations in rural topsoil in South Carolina: Potential for human health impact. *Science of the Total Environment,**402*, 149–156. 18538375 10.1016/j.scitotenv.2008.04.043 PMC2631568

[CR2] Aide, M.T., Aide, C. 2012. Rare earth elements: Their importance in understanding soil genesis. *ISRN Soil Sci* 783876.

[CR3] Aiuppa, A., Allard, P., D’Alessandro, W., Michel, A., Parello, F., Treuil, M., & Valenza, M. (2000). Mobility and fluxes of major, minor and trace metals during basalt weathering at Mt. Etna volcano (Sicily). *Geochimica Et Cosmochimica Acta,**64*, 1827–1841.

[CR4] Albanese, S., Ebrahimi, P., Aruta, A., Cicchella, D., De Vivo, B., & Lima, A. (2023). Potentially toxic elements in the soils of Campi Flegrei (south Italy) and the immediate surroundings: Spatial distribution, origin and probabilistic human health risk. *Chemosphere,**313*, 137297. 36410516 10.1016/j.chemosphere.2022.137297

[CR5] Ambrosino, M., Albanese, S., De Vivo, B., Guagliardi, I., Guarino, A., Lima, A., & Cicchella, D. (2022). Identification of rare earth elements (REEs) distribution patterns in the soils of Campania region (Italy) using compositional and multivariate data analysis. *Journal of Geochemical Exploration,**243*, 107112.

[CR6] Ambrosino, M., El-Saadani, Z., Abu Khatita, A., Mingqi, W., Palarea-Albaladejo, J., & Cicchella, D. (2023). Geochemical speciation, ecological risk and assessment of main sources of potentially toxic elements (PTEs) in stream sediments from Nile River in Egypt. *Water,**15*(13), 2308.

[CR7] Ambrosino, M., Palarea-Albaladejo, J., Albanese, S., & Cicchella, D. (2024). Mapping geochemical domains using stream sediment geochemistry: An approach based on compositional indicators in the Volturno River basin (South Italy). *Journal of Geochemical Exploration,**265*, 107545.

[CR8] Baize, D., & Sterckeman, T. (2001). Of the necessity of knowledge of the natural pedo-geochemical background content in the evaluation of the contamination of soils by trace elements. *Science of the Total Environment,**26*, 127–139. 10.1016/s0048-9697(00)00615-x 11213175

[CR9] Belon, E., Boisson, M., Deportes, I. Z., Eglin, T. K., Feix, I., Bispo, A. O., Galsomies, L., Leblond, S., & Guellier, C. R. (2012). An inventory of trace elements inputs to French agricultural soils. *Science of the Total Environment,**439*, 87–95. 23063913 10.1016/j.scitotenv.2012.09.011

[CR10] Beus, A. A. (1971). Titanium distribution in the lithosphere. *Chemical Geology,**8*(4), 247–275.

[CR11] Carr, M. H., & Turekian, K. K. (1961). The geochemistry of cobalt. *Geochimica et Cosmochimica Acta,**23*, 9–60.

[CR12] Catalano, R., Valenti, V., Albanese, C., Accaino, F., Sulli, A., Tinivella, U., Gasparo Morticelli, M., Zanolla, C., Giustiniani, M. (2013). Sicily’s fold-thrust belt and slab roll-back: The SI.RI.PRO. seis-mic crustal transect. J. Geol. Soc., London 170(3), 451–464.

[CR13] Chakhmouradian, A. R., & Wall, F. (2012). Rare earth elements: Minerals, mines, magnets (and more). *Elements,**8*, 333–340.

[CR14] Chen, J., & Yang, R. (2010). Analysis on REE geochemical characteristics of three types of REE-rich soil in Guizhou Province China. *Journal of Rare Earths.,**28*, 517–522.

[CR15] Cicchella, D., De Vivo, B., & Lima, A. (2005). Background and baseline concentration values of elements harmful to human health in the volcanic soils of the metropolitan provincial area of Napoli (Italy). *Geochemistry: Exploration, Environment, Analysis,**5*, 29–40.

[CR16] Cicchella, D., Zuzoloa, D., Albanese, S., Fedele, L., Di Tota, I., Guagliardi, I., Thiombane, M., De Vivo, B., & Lima, A. (2020). Urban soil contamination in Salerno (Italy): Concentrations and patterns of major, minor, trace and ultra-trace elements in soils. *Journal of Geochemical Exploration,**213*, 106519.

[CR17] Cidu, R., Vittori Antisari, L., Biddau, R., Buscaroli, A., Carbone, S., Da Pelo, S., Dinelli, E., Vianello, G., & Zannoni, D. (2013). Dynamics of rare earth elements in water-soil system: The case study of the Pineta San Vitale (Ravenna, Italy). *Geoderma,**193–194*, 52–67.

[CR18] Cressie, N. (1990). The origins of kriging. *Mathematical Geology,**22*, 239–252.

[CR19] Dantu, S. (2014). Spatial distribution and geochemical baselines of major/trace elements in soils of Medak district, Andhra Pradesh, India. *Environmental Earth Sciences,**72*, 955–981.

[CR20] Darnley, A. G., Björklund, A., Bølviken, B., Gustavsson, N., Koval, P., Plant, J. A., Steenfelt, A., Tauchid, M., Xie, X., Garret, R. G., & Hall, G. E. M. (1995). *A global geochemical database for environmental and resource management*. UNESCO Publishing.

[CR21] De Vivo, B., Lima, A., Bove, M. A., Albanese, S., Cicchella, D., Sabatini, G., Di Lella, L. A., Protano, G., Riccobono, F., Frizzo, P., & Raccagni, L. (2008). Environmental geochemical maps of Italy from the FOREGS database. *Geochemistry: Exploration, Environment, Analysis,**8*, 267–277.

[CR22] De Vivo, B., Lima, A., Catalano, G., & Chersicla, A. (1993). Detailed geochemical survey in the Peloritani Arc (Northeastern Sicily), Italy: Evidence of gold anomalies. *Journal of Geochemical Exploration,**46*, 309–324.

[CR23] D. Lgs. 152/2006. Norme in materia ambientale. Allegato 5, Parte IV, Tabella 1. Gazzetta Ufficiale n. 88 del 14 aprile 2006, Supplemento Ordinario n. 96. https://www.gazzettaufficiale.it/dettaglio/codici/materiaAmbientale. Accessed 12 Nov 2024.

[CR24] Deutsch, C. V., & Journel, A. G. (1998). *GSLIB*. Oxford University Press.

[CR25] Dongarrà, G., Manno, E., Sabatino, G., & Varrica, D. (2009). Geochemical characteristics of waters in mineralised area of Peloritani Mountains (Sicily, Italy). *Applied Geochemistry,**29*, 900–914.

[CR26] Efron, B., & Tibshirani, R. J. (1993). *An introduction to the bootstrap*. Chapman & Hall.

[CR27] Fabian, C., Reimann, C., Fabian, K., Birke, M., Baritz, R., Haslinger, E. The GEMAS Project Team, 2014. GEMAS: Spatial distribution of the pH of European agricultural and grazing land soil. Applied Geochemistry 48, 207-216.

[CR28] Ferla, P., & Omenetto, P. (2000). Metallogenic evolution of Peloritani Mountains (NE Sicily): A summary. *Mem. Soc. Geol. It.,**55*, 293–297.

[CR29] Force, E.R. 1976. *Geology and resources of titanium.* Geological Survey Professional Paper 959-A, B, C, D, E, F. Washington, D.C.: United States Government Printing Officehttps://pubs.usgs.gov/pp/0959a-f/report.pdf (PDF. Last accessed: March 3, 2025)

[CR30] Gałuszka, A. (2007). A review of geochemical background concepts and an example using data from Poland. *Environmental Geology,**52*, 861–870.

[CR31] Giacalone, A., Gianguzza, A., Dongarrà, G., Orecchio, S., Piazzese, D., Sciarrino, S., & Varrica, D. (2005). Metal distribution in the organic and inorganic fractions of soil: A case-study on soils from Sicily. *Chemical Speciation & Bioavailability,**17*, 8394.

[CR32] Gómez-Hernández, J. J., & Srivastava, R. M. (2021). One step at a time: The origins of sequential simulation and beyond. *Mathematical Geosciences,**53*, 193–209.

[CR33] Goovaerts, P. (1997). *Geostatistics for Naturale Resources*. Oxford University Press.

[CR34] Gruba, P., & Socha, J. (2016). Effect of parent material on soil acidity and carbon content in soils under silver fir (Abies alba Mill.) stands in Poland. *CATENA,**140*, 90–95.

[CR35] Helvaci, C., Stamatakis, M., Zagouroglou, C., & Kanaris, J. (1993). Borate minerals and related authigenic silicates in Northeastern Mediterranean Late Miocene continental Basins. *Exploration and Mining Geology,**2*, 14–22.

[CR36] IAEA- International Atomic Energy Agency, Vienna (Austria). 1989. *Uranium deposits in magmatic and metamorphic rocks.* Technical committee meeting on uranium deposits in magmatic and metamorphic rocks (https://inis.iaea.org/records/2pg2y-1dc63).

[CR37] Ion, A., & Cosac, A. (2023). Rare earth elements distribution in topsoil from Ditrău Alkaline Massif area, eastern Carpathians. *Romania. Heliyon.,**9*, 13976. 10.1016/j.heliyon.2023.e13976 PMC10006540 36915555

[CR38] Jarva, J., Tarvainen, T., Reinikainen, J., & Eklund, M. (2010). TAPIR —-Finnish national geochemical baseline database. *Science of the Total Environment,**408*, 4385–4395. 20673967 10.1016/j.scitotenv.2010.06.050

[CR39] Jenny, H. (1941). *Factors of Soil Formation*. McGraw-Hill.

[CR40] Jordens, A., Cheng, Y., & Waters, K. E. (2013). A review of the beneficiation of rare earth element bearing minerals. *Minerals Engineering,**41*, 97–114.

[CR41] Kottek, M., Grieser, J., Beck, C., Rudolf, B., & Rubel, F. (2006). World map of the Koppen- Geiger climate classification updated. *Meteorogische Zeitschrift,**15*, 259–263.

[CR42] Kubier, A., Wilkin, R. T., & Pichler, T. (2019). Cadmiun in soils and groundwater: A review. *Applied Geochemistry,**108*, 104388. 10.1016/j.apgeochem.2019.104388 PMC7147761 32280158

[CR43] Liguori, V., Brucculeri, R., 2004. *Groundwater management resources in Sicily, Italy.* http://balwois.com/balwois/administration/full_paper/ffp-546.pdf (accessed 11/12/2024).

[CR44] López, M., González, I., & Romero, A. (2008). Trace elements contamination of agricultural soils affected by sulphide exploitation (Iberian Pyrite Belt, Sw Spain). *Environmental Geology,**54*, 805–818.

[CR46] Manly, B. F. J. (1997). *Randomization, Bootstrap, and Monte Carlo Methods in Biology* (2nd ed.). Chapman Hall.

[CR47] Modabberi, S., Tashakor, M., Rajabian, N., Khorasanipour, M., Esmaeilzadeh, E., Ambrosino, M., & Cicchella, D. (2023). Characterization and chemical fractionation of potentially toxic elements in soils of a pre-mining mineralized area; an evaluation of mobility and environmental risk. *Environmental Geochemistry and Health,**45*, 4795–4815. 36941446 10.1007/s10653-023-01537-4

[CR48] Müller, G. (1969). Index of geoaccumulation in sediments of the RhineRiver. *Geoj,**2*, 108–118.

[CR49] Négrel, P., Sadeghi, M., Ladenberger, A., Reimann, C., Birke, M., The GEMAS Project Team. (2015). Geochemical fingerprinting and source discrimination of agricultural soils at continental scale. Chemical Geology *396*, 1–15.

[CR50] Nelson, D.W., Sommers, L.E., 1996. Total carbon, organic carbon, and organic matter. In: Sparks, D.L., et al., Eds., *Methods of Soil Analysis. Part 3. Chemical Methods*, SSSA Book Series No. 5, SSSA and ASA, Madison, WI, pp. 961–1010.

[CR51] Prashanth, L., Kattapagari, K. K., Chitturi, R. T., Baddam, V. R. R., & Prasad, L. K. (2015). A review on role of essential trace elements in health and disease. *Journal of Dr NTR University of Health Sciences,**4*, 75–85.

[CR52] QGIS Development Team. (2023). QGIS Geographic Information System (Version 3.34.6). Retrieved from https://qgis.org

[CR53] Rambeau, C. M. C., Baize, D., Saby, N., Matera, V., Adatte, T., & Föllmi, K. B. (2010). High cadmium concentrations in Jurassic limestone as the cause for elevated cadmium levels in deriving soils: A case study in Lower Burgundy France. *Environmental Earth Sciences,**61*, 1573–1585.

[CR54] Ramos, S. J., Dinali, G. S., Oliveira, C. D., Martins, G. C., Moreira, C. G., Siqueira, J. O., & Guilherme, L. R. (2016). Rare Earth Elements in the Soil Environment. *Current Pollution Reports,**2*, 28–50.

[CR55] Reimann, C., Birke, M., Demetriades, A., Filzmoser, P., O’Connor, P. (2014). Chemistry of Europe’s Agricultural Soils, Part B: General Background Information and Further Analysis of the GEMAS Data Set. Geologisches Jahrbuch Reihe B, Band B 103. Schweizerbart Science Publishers, Stuttgart.

[CR56] Reimann, C., & de Caritat, P. (2005). Distinguishing between natural and anthropogenic sources for elements in the environment: Regional geochemical surveys versus enrichment factor. *Science of the Total Environment,**337*, 91–107. 15626382 10.1016/j.scitotenv.2004.06.011

[CR57] Reimann, C., Fabian, K., Birke, M., Filzmoser, P., Demetriades, A., Négrel, P., Oorts, K., Matschullat, J., de Caritat, P., Albanese, S., Anderson, M., Baritz, R., Batista, M. J., Bel-Ian, A., Cicchella, D., De Vivo, B., De Vos, W., Dinelli, E., Ďuriš, M., … Sadeghi, M. (2018). GEMAS: Establishing geochemical background and threshold for 53 chemical elements in European agricultural soil. *Applied Geochemistry,**88*, 302–318.

[CR58] Reuter, H. I., Lado, L. R., Hengl, T., & Montanarella, L. (2008). Continental-scale digital soil mapping using European soil profile data: Soil pH. *Hamburger Beiträgezur Physischen Geographie und Landschaftsökologie,**19*, 91–102.

[CR59] Rollinson, H. R. (2014). *Using geochemical data: Evaluation, presentation, interpretation*. Routledge.

[CR60] Rosell, L., Orti, F., Kasprzyk, A., Playa, E., & Peryt, T. M. (1998). Strontium geochemistry of Miocene primary gypsum; Messinian of southeastern Spain and Sicily and Badenian of Poland. *Journal of Sedimentary Research,**68*, 63–79.

[CR61] Rutkowski, P., Diatta, J., Konatowska, M., Andrzejewska, A., Tyburski, Ł, & Przybylski, P. (2020). Geochemical referencing of natural forest contamination in Poland. *Forests,**11*, 157.

[CR62] Salminen, R., Bogatyrev, I., Chekushin, V., Glavatskikh, S. P., Gregorauskiene, V., Niskavaara, H., Selenok, L., Tenhola, M., & Tomilina, O. (2004). Geochemical baselines of nickel and chromium in various surficial materials in the Barents Region, NW Russia and Finland. *Geostandards and Geoanalytical Research,**28*, 333–341.

[CR63] Salminen, R., & Gregorauskiene, V. (2000). Considerations regarding the definition of a geochemical baseline of elements in the surficial materials in areas differing in basic geology. *Applied Geochemistry,**15*, 647–653.

[CR64] Santos-Francés, F., Martinez-Grana, Ã., Alonso Rojo, P., & García, S. A. (2017). Geochemical background and baseline values determination and spatial distribution of heavy metal pollution in soils of the Andes Mountain Range (Cajamarca Huancavelica, Peru). *International Journal of Environmental Research and Public Health,**14*, 859. 28788105 10.3390/ijerph14080859 PMC5580563

[CR65] Sappa, G., Barbieri, M., & Andrei, F. (2020). Assessment of trace elements natural enrichment in topsoil by some Italian case studies. *SN Appl. Sci.,**2*, 1409.

[CR66] Sierra, C., Ordóñez, O., Saavedre, A., & Gallego, J. R. (2015). Element enrichment factor calculation using grain-size distribution and functional data regression. *Chemosphere,**119*, 1192–1199. 25460761 10.1016/j.chemosphere.2014.10.024

[CR67] Singh, A., Maichle, R. (2015). ProUCL Version 5.1 User Guide. U.S. Environmental Protection Agency, Washington, DC. (EPA/600/R-07/041). https://www.epa.gov/land-research/proucl-software

[CR68] Slessarev, E. W., Lin, Y., Bingham, N. L., Johnson, J. E., Dai, Y., Schimel, J. P., & Chadwick, O. A. (2016). Water balance creates a threshold in soil pH at the global scale. *Nature,**540*, 567–569. 27871089 10.1038/nature20139

[CR69] SNPA – Sistema nazionale per la protezione ambientale. *Linea guida per la determinazione valori di fondo per i suoli e per le acque sotterranee*. (accessed 11/12/2024). https://www.isprambiente.gov.it/files2018/pubblicazioni/manuali-linee-guida/MLG_174_18.pdf

[CR70] Storer, D. A. (1984). A simple high sample volume ashing procedure for determining soil organic matter. *Communications in Soil Science and Plant Analysis,**15*, 759–772.

[CR71] Suresh, G., Sutharsan, P., Ramasamy, V., & Venkatachalapathy, R. (2012). Assessment of spatial distribution and potential ecological risk of the heavy metals in relation to granulometric contents of Veranam lake sediments India. *Ecotoxicol. Environ. Saf.,**84*, 117–124. 22835728 10.1016/j.ecoenv.2012.06.027

[CR72] Tack, F. M., Vanhaesebroeck, T., Verloo, M. G., Van Rompaey, K., & Van Ranst, E. (2005). Mercury baseline levels in Flemish soils (Belgium). *Environmental Pollution,**134*, 173–179. 15572235 10.1016/j.envpol.2004.05.031

[CR73] Tarvainen, T., & Paukola, T. (1998). Use of geochemical databases to delineate risk areas for contaminated groundwater. *Journal of Geochemical Exploration,**64*, 177–184.

[CR74] Tomlison, D. L., Wilson, J. G., Harris, C. R., & Jeffrey, D. W. (1980). Problems in the assesment of heavy-metal levels in estauries and the formation of a pollution index. *Helgoläander Meeresuntersuchungen,**33*, 566–575.

[CR75] US EPA. (1992). *Common chemicals found at Superfund sites US Government.* Printing Office, Washington, DC. EPA 540/R-94/044.

[CR76] Usero, J., Garcia, A., Fraidias, J. (2000) *Andalucia board, environmental counseling*. 1st Edn., Seville, Editorial.

[CR77] Varrica, D., Lo Medico, F., Zuccolini, M. V., Miola, M., & Alaimo, M. G. (2024). Geochemical baseline values determination and spatial distribution of trace elements in topsoils: An application in Sicily region (Italy). *Science of the Total Environment,**955*, 176951. 39426544 10.1016/j.scitotenv.2024.176951

[CR78] Wang, X., Zhang, Q., & Zhou, G. (2007). National-scale geochemical mapping projects in China. *Geostandards and Geoanalytical Research,**31*, 311–320.

[CR79] Xie, X., & Ren, T. (1993). National geochemical mapping and environmental geochemistry—progress in China. *Journal of Geochemical Exploration,**49*, 15–34.

[CR80] XLSTAT (versione 2023.3). Addinsoft, Parigi, Francia. https://www.xlstat.com/en/.

[CR81] Yongming, H., Peixuan, D., Junji, C., & Posmentier, E. S. (2006). Multivariate analysis of heavy metal contamination in urban dusts of Xi’an. *Central China. Sci. Total Environ.,**355*, 176–186. 15885748 10.1016/j.scitotenv.2005.02.026

[CR82] Zhang, F. S., Yamasaki, S., & Kimura, K. (2001). Rare earth element content in various waste ashes and the potential risk to Japanese soils. *Environment International,**27*, 393–398. 11757853 10.1016/s0160-4120(01)00097-6

[CR83] Zuzolo, D., Cicchella, D., Lima, A., Guagliardi, I., Cerino, P., Pizzolante, A., Thiombane, M., De Vivo, B., & Albanese, S. (2020). Potentially toxic elements in soils of Campania region (Southern Italy): Combining raw and compositional data. *Journal of Geochemical Exploration,**213*, 106524.

